# Editorial: Oxidative Stress Revisited—Major Role in Vascular Diseases

**DOI:** 10.3389/fphys.2019.00788

**Published:** 2019-06-21

**Authors:** Cristina M. Sena, Raquel Seiça, George Perry

**Affiliations:** ^1^Institute of Physiology, Faculty of Medicine, University of Coimbra, Coimbra, Portugal; ^2^Department of Biology, University of Texas at San Antonio, San Antonio, TX, United States

**Keywords:** oxidative stress, inflammation, aging, non-communicable diseases, novel therapeutic approaches

Oxidative stress is an underlying factor in health and disease (Sies, 1985). The concept of oxidative stress was formulated in 1956 by Harman, and throughout the years substantial knowledge established that oxidative stress, an imbalance between oxidants and antioxidants with a disruption of redox signaling and control, is crucial for vascular diseases (Harman, 1956).

In this Frontiers Research topic oxidative stress was revisited by discussing old paradigms and suggesting new therapeutic approaches to decreased oxidative stress and reduce the progression of vascular diseases.

In the vasculature, several sources promote an increment in oxidative stress. Free radicals (such as the superoxide anion radical, hydrogen peroxide, hydroxyl radical, as well as the nitric oxide radical and peroxynitrite) are major sources of oxidative stress (Sies, 1985). Vascular oxidative stress promotes endothelial dysfunction and atherosclerosis progression (Sena et al.) playing a major role in several vascular diseases including neurodegenerative diseases (Carvalho and Moreira) and diabetic complications (Santiago et al.; Sena et al.; Teodoro et al). This was elegantly discussed by several authors in this research topic.

Oxidative stress may have a critical role in the neurovascular uncoupling underlying brain aging and dysfunction. Carvalho and Moreira discussed how oxidative stress is in the front line of vascular alterations observed in brain aging and neurodegenerative conditions, particularly Alzheimer disease.

In addition, Lourenço et al. demonstrated that non-pathological brain aging involves changes in both neurovascular and neurometabolic function in the hippocampus, in close correlation with compromised cognitive function, suggesting a role for oxidative stress. This data supports an impairment of neurovascular response in connection with cognition decline due to oxidative environment-dependent compromised nitric oxide (NO) signaling from neurons to vessels during aging.

Oxidative stress and the potential role of reactive oxygen species in the initiation and progression of systemic sclerosis vasculopathy was reviewed by Abdulle et al. These authors suggest the use of oxidative stress related read-outs as clinical biomarkers of disease activity and evaluate potential anti-oxidative strategies in systemic sclerosis (Abdulle et al).

The synergistic contributions of redox-inflammatory processes for endothelial dysfunction in diabetic retinopathy were discussed by Santiago et al. with emphasis in the endothelial cell communication with other retinal cells.

The role of reactive oxygen species in the development of pulmonary vasculature changes under hypoxic conditions was discussed by Siques et al.

In different chapters, authors suggest and discuss some therapeutic strategies to protect the vasculature against oxidative stress. Therapeutic approaches such as policosanol consumption and melatonin have been investigated and discussed. The physiological policosanol consumption resulted in significant lowering of brachial and central aortic blood pressure in a dose-dependent manner accompanied by an improvement in lipid profile resulting in improved anti-oxidant and anti-glycation activities in healthy Korean subjects with pre-hypertension (Kim et al). Melatonin and its therapeutic potential was discussed by Guo et al. on cerebral autoregulation following subarachnoid hemorrhage.

Environmental stress factors are important both in adults and in early life. Exposure to stressful environments produced persistent increases in plasma corticosterone and reductions of brain and cardiac NO production followed by a delayed decrease in the NO-dependent component of endothelium-dependent relaxation—changes that collectively accelerated blood pressure increases only in young borderline hypertensive rats (Bernatova et al.). In addition, due to the role of macrophages in all stages of the inflammatory response by acquiring distinct functional phenotypes that are directed by the tissue type and environmental cues, Takahashi et al. highlighting the anti-inflammatory effects of AM18W-13a extract to reduce inflammation in murine macrophages.

Importantly, environmental stress factors during fetal and perinatal life can shape the future health of the individual and increase susceptibility to several adult diseases. Suboptimal intrauterine conditions induce alterations in placental redox balance, associated with poor fetal development and low birth weight. Rodríguez-Rodríguez et al. summarize data on specific redox alterations in key cardiovascular control organs induced by exposure to known stress factors in experimental animals and discuss the emerging role of the mitochondria.

This Frontiers Research Topic in oxidative stress is a view into the rich places of research that have yet to be tapped relative to understanding the role oxidative stress plays both in health and disease.

We are grateful to our contributors for sharing their important work.

## Author Contributions

CS drafted the editorial. RS and GP read and modified the editorial.

### Conflict of Interest Statement

The authors declare that the research was conducted in the absence of any commercial or financial relationships that could be construed as a potential conflict of interest.
